# Management of hypertension and multiple risk factors to enhance cardiovascular health in Singapore: The SingHypertension cluster randomized trial

**DOI:** 10.1186/s13063-018-2559-x

**Published:** 2018-03-14

**Authors:** Tazeen H. Jafar, Ngiap Chuan Tan, John C. Allen, Eric A. Finkelstein, Paul Goh, Peter Moey, Joanne Hui Min Quah, Siew Wai Hwang, Juliana Bahadin, Anandan Gerard Thiagarajah, Jason Chan, Gary Kang, Agnes Koong

**Affiliations:** 1 Program in Health Services & Systems Research, Duke–NUS Medical School, Singapore, Singapore; 2 Department of Renal Medicine, Singapore General Hospital, Singapore, Singapore; 3 Duke Global Health Institute, Duke University, Durham, North Carolina USA; 4 SingHealth Polyclinics, Singapore, Singapore; 5 Health Services Research Centre, SingHealth, Singapore, Singapore; 6 Centre for Quantitative Medicine, Office of Clinical Sciences, Duke–NUS Medical School, Singapore, Singapore

**Keywords:** Hypertension, Blood pressure, cardiovascular, Single-pill combination, Motivational counseling, Telephone follow-up

## Abstract

**Background:**

Hypertension is a serious public health problem in Singapore and is associated with significant morbidity and mortality from cardiovascular disease (CVD) with considerable implications for health-care resources. The goal of the trial is to compare a multicomponent intervention (MCI) to usual care to evaluate the effectiveness and cost-effectiveness of the MCI for lowering blood pressure (BP) among adults with uncontrolled hypertension in Singapore primary-care clinics.

**Methods/design:**

The study is a cluster randomized trial in eight polyclinics in Singapore: four deliver a structured MCI and four deliver usual care. The components of the MCI are: (1) an algorithm-driven antihypertensive treatment for all hypertensive individuals using single-pill combination (SPC) and lipid-lowering medication for high-risk hypertensive individuals, (2) a motivational conversation for high-risk hypertensive individuals, (3) telephone-based follow-ups of all hypertensive individuals by polyclinic nurses, and (4) discounts on SPC antihypertensive medications.

The trial will be conducted with 1000 individuals aged ≥ 40 years with uncontrolled hypertension (systolic BP ≥ 140 mmHg or diastolic BP ≥ 90 mmHg, based on the mean of the last two of three measurements) in eight polyclinics in Singapore. The primary outcome is change in systolic BP from baseline to follow-up at 24 months post-randomization. The incremental cost of MCI per CVD disability adjusted life years (DALY) averted and quality adjusted life years (QALY) saved will be computed.

**Discussion:**

The demonstration of an effective and cost-effective hypertension control program that is implementable in busy polyclinics would provide compelling evidence for upscaling the program across all primary-care centers in Singapore, and possibly other regional countries with a similar health-care structure.

**Trial registration:**

Clinicaltrials.gov, NCT02972619. Registered on 23 November 2016.

**Electronic supplementary material:**

The online version of this article (10.1186/s13063-018-2559-x) contains supplementary material, which is available to authorized users.

## Background

Cardiovascular disease (CVD) accounted for one-third of all deaths in 2015, and trend data from the past decade indicate that Singapore lags behind in age-standardized rates of decline in CVD death rates compared to other high-income countries [1]. High blood pressure (BP) confers the greatest attributable risk of death, and is responsible for substantial disability from stroke, myocardial infarction, heart failure, and kidney disease, thereby consuming considerable amounts of health-care expenditure [2]. Currently, over 1 billion people have uncontrolled BP globally, and this number is expected to increase to 1.5 billion by 2030 [1].

The relationship between uncontrolled BP and stroke has been shown to be steeper in Asian populations, and therefore, the implications of uncontrolled BP are graver in Southeast Asia [2]. According to the 2010 National Health Survey Singapore, one in four adults aged 30 years or older suffered from hypertension [3]. Of these, about 50% had uncontrolled BP, and one-fourth were not on antihypertensive medication. Immediate health system interventions are, therefore, needed.

Evidence from a systematic review of quality improvement health system strategies to control BP (self-monitoring, organization of care, educational initiatives directed at patients or physicians, nurse- or pharmacist-led care, and automated appointment reminders) shows the clear benefit of organized or structured care compared to single interventions [4]. More recently, one of the best practices identified in the insured U.S. population included a hypertension management program with components including single-pill combination (SPC) antihypertensive agents and medical assistant visits for measurement of BP, which a demonstrated beneficial impact on BP control [5]. However, there is a paucity of empirical trial evidence on strategies to enhance the effectiveness of comprehensive care for hypertension and other chronic conditions in Singapore and countries with a similar health-care infrastructure [6].

The majority (60%) of individuals with hypertension or diabetes in Singapore seek care at government primary-care clinics (or polyclinics) [3]. The remaining 40% of .individuals seek care from over 2000 private general practitioners. There are 18 polyclinics in Singapore staffed by about 400 government general practitioners and family physicians managed by two major administrative health-care groups (nine each by the SingHealth and the National Healthcare groups) located across geographic areas of Singapore. Each polyclinic has a basic laboratory and a pharmacy, and the services are subsidized for Singaporeans and permanent residents.

Recently, we reported findings from a pilot feasibility trial in two polyclinics in Singapore with 100 adults with uncontrolled hypertension. The purpose of the pilot study was to assess the feasibility of intervention implementation in polyclinics with regards to the fidelity of the main interventional components by using the polyclinic workforce and infrastructure to inform the design and scalability of a future full-scale cluster randomized controlled trial. The pilot study also sought feedback from stakeholders. Findings from the pilot study have been incorporated in the full-scale cluster randomized controlled trial protocol, which will evaluate the fidelity, effectiveness, and cost-effectiveness of the intervention versus usual care. The current protocol paper describes the full-scale cluster randomized controlled trial, SingHypertension, in detail.

## Methods/design

We will evaluate the effectiveness of a multicomponent intervention (MCI), SingHypertension, compared to usual care in lowering systolic blood pressure (SBP) over 2 years among 1000 individuals aged 40 years or older with previously diagnosed hypertension and uncontrolled BP (SBP ≥140 mmHg or diastolic blood pressure (DBP) ≥90 mmHg based on the mean of the last two of three measurements) in eight government clinics in Singapore. Four clinics will deliver a structured SingHypertension MCI and four will deliver usual care.

The SingHypertension MCI consists of the following four components: (1) an algorithm-driven antihypertensive treatment for all individuals using SPC and lipid-lowering medication for high-risk individuals, (2) a motivational conversation for high-risk individuals, (3) telephone follow-ups of all hypertensive individuals by polyclinic nurses, and (4) discounts on SPC antihypertensive medications. The comparator is usual care in the polyclinics.

The main aims of the trial are:

1. To determine whether the effectiveness of the structured multicomponent program described above in 1–4 is better than usual care in improving the primary outcome of SBP and the secondary outcome of cardiovascular risk factors compared to usual care in the polyclinics in Singapore.
2. To quantify the source of payment and costs for the MCI and perform an incremental cost-effectiveness analysis in terms of cost per projected disability adjusted life year (DALY) averted and quality adjusted life year (QALY) saved from the societal, payer, and participant perspectives relative to usual care.

An additional aim is:

3. To determine the impact of the above-mentioned program on adherence to antihypertensive and lipid-lowering medications.

### Hypotheses

In polyclinics in Singapore:

- The structured, multicomponent primary-care program is better than the usual care for improving SBP among hypertensive individuals with uncontrolled hypertension.
- The structured multicomponent program is cost-effective relative to usual care in terms of incremental cost per projected DALYs averted and QALYs saved from the societal, payer, and participant perspectives.

### Trial design

The study is a cluster randomized controlled trial in eight polyclinics in Singapore conducted according to the framework of the U.K. Medical Research Council (MRC) for implementing complex intervention trials [5]. A cohort of 125 individuals with uncontrolled hypertension will be recruited in each clinic and followed over a 2-year period for change in BP.

**Fig. 1:**
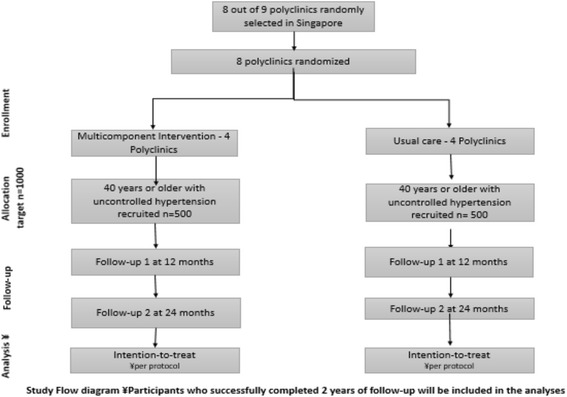
Study flow diagram

A Standard Protocol Items: Recommendations for Interventional Trials (SPIRIT) checklist is provided as Additional file 1. The flow diagram for the study protocol is included as Fig. 1.

### Trial setting and randomization

As mentioned above, the outpatient health-care system of Singapore is serviced by 18 subsidized government polyclinics (nine each by the SingHealth and the National Healthcare administrative network groups) staffed by about 400 government general practitioners. The trial will be conducted in eight (of nine) SingHealth Polyclinics and will receive intervention or usual care. The trial was planned to be conducted in eight of nine clinics under the SingHealth group.

The initial randomization was generated by a computer code such that four clinics are randomized to intervention or usual care. One clinic (MP) was excluded to maintain the balanced randomization. Moreover, one SingHealth clinic (GL) previously included in the feasibility study as an intervention clinic was pre-allocated to intervention to minimize contamination. Thus, masked randomization was performed for seven clinics and open allocation (GL) for one clinic. However, due to an administrative restructuring of the polyclinics in Singapore, GL is no longer under SingHealth. Thus, Geylang (GL) was replaced by Marine Parade (MP) as one of the four intervention clinics.

### Inclusion criteria

1. All individuals aged 40 years or older
2. Singapore citizen or permanent resident
3. Visited the recruiting polyclinic at least twice during the last 12 months
4. Individuals with a diagnosis of hypertension (SBP ≥ 140 mmHg or DBP ≥ 90 mmHg on two or more prior visits, physician-diagnosed hypertension, or on antihypertensive medication) and uncontrolled blood pressure (SBP ≥ 140 mmHg or DBP ≥ 90 mmHg)

### Exclusion criteria

1. Active systemic illness including fever or recent hospitalization (i.e., during the last 4 months)
2. Clinically unstable heart failure or advanced kidney disease (estimated CKD-Epi glomerular filtration rate < 40 ml/min/1.73 m^2^ or nephrotic range proteinuria, i.e., 3 g/d or more)
3. Known liver disease
4. Pregnant or breastfeeding
5. Any other major debilitating disease or mental illness that precludes the validity of informed consent [4]

### Intervention

The intervention is a structured multicomponent primary-care program (MCI) comprising:

1. An algorithm-driven antihypertensive treatment for all hypertensive individuals using SPC and lipid-lowering medication for high-risk hypertensive individuals
  The treatment algorithm has been prepared by a team of nephrologists, cardiologists, and pharmacologists in consultation with SingHealth primary-care physicians and the Clinical Cardiovascular Work Group. The provider orientation and training curriculum will be developed by the same team in conformity with relevant international guidelines, using the case method, aiming for a global cardiovascular risk reduction [5]. In addition to advice on behavior change (diet, physical activity, moderation of alcohol intake, and smoking cessation), the focus will be on target BP and LDL cholesterol levels using medications, per treatment algorithm.
  The treatment algorithm is described in Additional file 2. For all high-risk hypertensive individuals, the SPC with angiotensin II receptor blocker (ARB) and a diuretic will be initiated at half-standard dose of each, and titrated to full dose if BP remains uncontrolled. These combination regimens have been shown to be effective and safe in lowering BP and preventing CVD in trials, [6, 7] and are used across public health general practices in Canada [8]. Moreover, both angiotensin-converting-enzyme inhibitor (ACEI) plus diuretic and ARB plus diuretic are in the drug formulary in the public-sector hospitals in Singapore. High-risk hypertensive individuals will also be initiated on lipid-lowering therapy with statins (already in the formulary in the polyclinics) aiming for LDL-C < 2.6 mmol/L (< 100 mg/dl), as per the algorithm [9]. The first-line antihypertensive for individuals at medium or low risk will be calcium channel blockers, the next agents would be ACEIs (preferably in those less than 55 years) or thiazide-type diuretics (preferably in those aged 55 years and older) initiated at half-standard dose, and up-titrated as necessary [10]. However, if BP is greater than 20/10 mmHg above target at a diagnostic visit, SPC therapy would be initiated, in line with the JNC-8 recommendation [11]. The target BP will be <140/90 mmHg. However, the target BP for hypertensive individuals with proteinuria or pre-existing CVD will be < 130/80 mmHg. The aim is to avoid lowering DBP < 60 mmHg, especially for older adults with coronary artery disease. Home BP readings are expected to be 10 mmHg lower and advice on titration will be adjusted accordingly, subject to use of a calibrated home BP monitoring device [12].
  All individuals with a Framingham CVD score indicating a risk of acute coronary heart disease (CHD) of 20% or more over 10 years, or with diabetes, target organ damage, or pre-existing CVD will be categorized as high risk. All other hypertensive individuals will be categorized as low/medium risk. The polyclinics have an existing adjustment factor in the equation to account for the local Malay and Indian population, which will be used as is. After CVD risk assessment at triage and measurement of BP by nurses, the hypertensive individuals will be triaged to physicians for a further evaluation of CVD risk with the checklist for high CVD risk with a total contact time of about 5–10 min, including prescription of antihypertensive medications per treatment algorithm.
  The use of home BP monitors (upper arm Omron digital device), which are part of usual practice, will be encouraged and devices will be calibrated in the clinic every 6 months (by comparing concomitantly obtained office readings).
  Hypertensive individuals initiated on ACEI or ARB will be given a laboratory request for measurement of their renal panel (serum sodium, potassium, and creatinine) 4 weeks after treatment initiation with a report fed back to the nurse. Lab results with a change of 20% or more than normal level will be flagged for potential action, such as a change in class of medication to calcium channel blockers with or without diuretics (the direct reporting system from laboratory to providers is already in practice at the polyclinics). Hypertensive individuals initiated on statins will be given a laboratory request for serum alanine transaminase and muscle creatinine kinase in 4 weeks with a report fed back to the nurse, which will be flagged by the laboratory if the results are abnormal. If the enzyme levels are more than three times the upper limit of normal, statins will be discontinued. If the levels are less than three times the upper limit and more than the normal limit, the levels will be measured again in another 4 weeks.
  Measures will be taken to ensure there is adequate supply of all antihypertensive medications. SPC medications (currently in the formulary at public-sector hospitals at discounted rates) will be made available at the polyclinics at the same prices.
  All physicians in the polyclinics randomized to the intervention will be invited for training. These providers will be intensively trained in the management and treatment algorithms for hypertension using non-pharmacologic therapy and combination antihypertensive regimen drug intervention. The training sessions for physicians will be conducted by a nephrologist (a principal investigator) and scheduled during regular continued medical education hours for the convenience of providers. The providers will also be trained in completing a physician management checklist after evaluating and managing patients as per the study treatment algorithm (Additional file 3). The management of other conditions and diseases will continue at the discretion of physicians and protocols at the polyclinic, with recommended referrals as necessary.
  A follow-up session will be conducted at each intervention clinic as part of the trial management meeting after recruitment of the first 20–30 patients to clarify any questions related to implementation of the study algorithm.
2. Motivational conversation for high-risk hypertensive individuals
  A motivational conversation curriculum will be prepared for hypertensive patients. This has been prepared by a team of specialist psychologists skilled in communication in health care in consultation with a principal investigator (a nephrologist). Nurses in the intervention clinics will be taught motivational conversation techniques by a specialist psychologist skilled in communication in health care [13, 14]. The counseling approach of the motivational conversation is intended to help hypertensive individuals resolve problems and make decisions, to encourage participation, to give empowerment, to help prioritize, and to set goals for self-care and medication adherence [14].
  Intensive training will be conducted with study roll-out with a shorter retraining session (1 day) after 1 year.
  For high CVD risk hypertensive individuals, a motivational conversation session will be conducted by the trained nurse. The initial motivational conversation session with the patient is anticipated to last between 15 and 20 min. These contact times fit within the existing work schedule and patient load at the clinics.
3. Telephone-based follow-ups of all hypertensive individuals by polyclinic nurses
  All hypertensive individuals will be followed up for self-monitoring of BP over telephone by the nurse with a telephone follow-up checklist (Additional file 4), and receive advice on adherence to treatment by the nurse every 4 weeks for 3 months, and then every 3 months for the duration of the project (2 years). Information on adverse events will also be obtained and the action plan [update the clinical research coordinators (CRCs) and discontinue the suspected drug with doctor’s advice and arrange visit to clinic as appropriate] will be communicated to participants. The telephone appointments will be scheduled in advance and recorded in the central electronic medical record system of the polyclinics, and automated reminders will be sent to alert the nurse about the telephone appointment. The frequency of follow-up clinic visits will be determined by the level of BP control.
  Hypertensive individuals will visit the clinic every 6–8 weeks until their BP is controlled to target, and then every 3–4 months. More frequent visits will be scheduled for participants with any symptoms or adverse events or those reporting very high BP (>180/110 mmHg). Scheduled meetings will be arranged among physicians and nurses to review cases that have not been able to achieve the target, and an appropriate action plan will be communicated.
4. Discounts on SPC antihypertensive medication
  The SPC ARB/hydrochlorothiazide antihypertensive will be discounted at 50% in the trial for the participants in the intervention group for whom the medication is prescribed.

### Usual care

The health providers in the four polyclinics randomized to usual care will continue their existing practices. Hypertensive individuals will continue to pay for the services (i.e. physician or nurse consultation, and any diagnostics or medications) as per their existing model of reimbursement.

#### Training of research staff

The CRCs will be trained on all study-related measurements, standard operating procedures, and filling out study questionnaires. Systematic post-training competency will be assessed for the CRCs on study-related measurements and the informed consent process prior to them being deployed to the polyclinics.

#### Site initiation meetings

Before the study roll-out, site initiation meetings led by a principal investigator will be scheduled in each polyclinic, to be attended by the site principal investigator, physicians, nurses, pharmacists, lab staff, and CRCs. The site initiation meetings included a presentation on the trial and Q&A session with the study site team.

### Screening assessment

In the polyclinics, all hypertensive individuals undergo computerized CVD risk scoring (using the Framingham CVD score) at triage, and those with hypertension get a panel of fasting blood and urine tests at subsidized cost at the time of initial diagnosis and then annually. The CRCs will approach individuals visiting the polyclinic to identify those with physician-diagnosed hypertension with an approach that fits each clinic’s work flow, including at the health monitoring station. They will also approach individuals presenting at the lab for their annual panel tests. These individuals will be invited to be screened for eligibility in the trial with the pre-screening form. During the pre-screening process, the CRC will measure BP three times with 3-minute intervals between readings, in a sitting position with arm rested using an upper arm calibrated automated Omron device™ (HEM-7130) to identify individuals with uncontrolled high BP. The average of the last two BP measurements will be calculated. Individuals with average SBP ≥ 140 mmHg or DBP ≥ 90 mmHg will be considered to have uncontrolled high BP in the pre-screen.

A total of 1000 participants will be recruited: 125 from each of the eight polyclinics, targeting a recruitment rate of two or three hypertensive individuals per clinic per day for about 9 months. Recruitment will be in batches of four and four clinics sequentially (balanced by randomized group). Following the pre-screening stage, the CRC will obtain informed consent from individuals who are considered eligible based on the pre-screen. After obtaining the written informed consent, the CRC and trained physician will screen the hypertensive individuals for eligibility with the screening form and review their medical records and history to confirm the diagnosis of hypertension. The CRC will also administer questionnaires to record the baseline BP readings measured in the pre-screening and to collect anthropometric measurements (weight, height, and waist circumference) and other baseline data for information on socio-demographics (age, gender, ethnicity, religion, education, marital status, employment status, housing, and personal and household income), co-morbidities, dietary (validated food frequency questionnaire), lifestyle (international physical activity questionnaire), and tobacco use, direct and indirect health-care cost (payments on consultation, laboratory tests, and medications), and quality of life (EQ-5D-5 L). Routine panel tests (the hypertensive panel or the diabetes panel if the patient has both physician-diagnosed hypertension and diabetes at baseline) for enrolled participants will be collected by the CRCs. Besides the routine panel tests collected at the clinic, further tests will be performed for eligible persons at baseline, including urine spot tests (urine spot albumin, sodium, and creatinine). Routine panel tests and urine spot tests can be performed within a 2-week window (±2 weeks) of the baseline visit.

The BP measurements performed as part of pre-screening will be used as the baseline BP measurements in the study. Subjects will be instructed not to drink tea or coffee for at least 30 min prior to BP readings. All measurements will be during the first half of the day to avoid nocturnal dipping of BP.

### Outcomes assessment

All hypertensive individuals will be assessed at baseline, 1 year (interim visit), and 2 years (final visit) after randomization by trained outcome assessors who are independent to the intervention. Additional telephone follow-ups by outcome assessors will be used to track for missing data if necessary. In addition, the outcome assessors will also call the hypertensive individuals by telephone at 4-month intervals when a follow-up questionnaire will be administered to collect data on recommended life-style changes (tobacco use and physical activity pattern), self-care (home BP monitoring), and medication use (including herbal and traditional Chinese). Hypertensive individuals will be asked to keep a record of the number of visits to their general practitioners during which their BP was measured, transport costs, general practitioner prescriptions, diagnostics, drugs purchased and currently used, any changes in drugs, and hospital visits. In addition, details will also be collected from the hypertensive individuals on any emergency room visits, chest pain, transient ischemic attack or new onset stroke, serious medication side effects, or contact with health professionals. Information on hospitalizations will also be obtained included the reason for hospitalization and the costs incurred. This information will be also be tracked through central computerized registries. The information on drugs will also be extracted through pharmacy dispensing records. Hospital records will be reviewed and events adjudicated by cardiologists and neurologists masked to randomization status, using guidelines recommended by the American Heart Association. Data on mortality from the death registry will be tracked during the follow-up period.

The outcome assessors (CRCs) will extract process and outcome measures from the polyclinic general practitioners’ and nurses’ notes. These will include number and duration of contact with the patient (whether in person or by phone), changes in prescribed medication and dose, and reports of any adverse events from medications. To clarify, the CRCs will collect information on medication change at scheduled baseline, interim, and 2-year clinic visits as well as during routine clinic visits. Access to patient clinical data will be facilitated by a polyclinic staff member. In addition, a questionnaire on FFQ (food frequency questionnaire), IPAQ (International physical activity questionnaire), tobacco use, and EQ-5D (Euro-Qol-5D) will be administered to all participants and BP (as in baseline) will be measured at the interim and final follow-up visits. LDL cholesterol levels, spot urine albumin to creatinine ratios, and spot urine sodium will be measured at the final follow-up visit.

CRCs for usual care will not be assigned at intervention clinics and vice versa.

Hypertensive individuals will be given a S$5 travel voucher at baseline and a S$20 travel voucher for the 1-year and 2-year visits, and will be reimbursed for the full cost of research-related laboratory tests. All participants from polyclinics assigned to intervention or usual care will continue to pay for all services as per their existing model of care. However, the SPC antihypertensive regimen recommended in the study algorithm will be subsidized at 50% of the cost by the project.

### Analysis

#### Primary outcomes measure

The primary outcome will be any change in SBP from baseline to the final follow-up at 2 years post-randomization.

#### Secondary outcomes measures

1. Change in serum LDL cholesterol from baseline to final follow-up at 2 years post-randomization
2. Proportion of hypertensive individuals with BP controlled to target or a > 5 mmHg decrease in SBP
3. Proportion of hypertensive individuals with a decrease in LDL cholesterol of > 0.4 mmol/L (> 15 mg/dl)
4. Composite outcome of death or hospital admission due to CHD, heart failure, or stroke
5. Individual outcomes of all-cause mortality, CVD deaths, or hospital admission due to CHD, heart failure, or stroke
6. A decrease of > 0.5% in glycated hemoglobin or change in proportion of hypertensive individuals with diabetes with glycated hemoglobin < 7%
7. Change in albuminuria from baseline to follow-up
8. Change in estimated CKD-EPI glomerular filtration rate from baseline to follow-up
9. Change in cardiovascular risk score
10. Individual outcomes of a change in (a) DBP and (b) total cholesterol from baseline to end of follow-up at 2 years
11. Change in lifestyle (diet or physical activity based on self-report) or body mass index between groups
12. Change in SBP from baseline to 1-year post-randomization
13. Change in serum LDL cholesterol from baseline to 1-year post-randomization

### Statistical analysis

Throughout, *p* < 0.05 will be considered statistically significant. Continuous variables will be summarized as mean (standard deviation) or median (interquartile range) as appropriate and categorical variables will be summarized as number (percentage). All analyses will be performed in accordance with the intention to treat principle.

The effectiveness of the structured intervention package compared to usual care will be assessed in the context of linear or logistic linear mixed effects models, depending on whether the endpoint is continuous or binary. In particular, changes in SBP at 2 years post-randomization (primary endpoint) will be compared between the treatment and usual care arms in the context of a linear mixed effects model with random effects for polyclinic, patient, and treatment effect nested in patient and fixed effects for time and treatment effect at follow-up. In these models, the test of treatment effect at follow-up is the test of the treatment’s effectiveness. Note that the test of treatment effect at follow-up is equivalent to a test for a difference in slopes of SBP change between the treatment and control groups.

While randomization ensures there is an approximate balance with respect to both known and unknown confounders, tests of treatment effectiveness will also be performed after adjustment for potential covariates including age, sex, socio-demographic variables, and ethnicity. These potential confounders will be selected based on stepwise variable selection with the Akaike information criterion. Further, while attrition is not expected to be extremely high, mixed effects models are able to incorporate all non-missing data with no need for imputation and they remain unbiased if the data missingness pattern, conditional on the observed data and covariates, does not depend on the values of the missing data. That is, the data is missing at random [15]. However, if the pattern of data missingness is related to covariates, then these covariates will be included as fixed effects in each of the models described above. Further, as a sensitivity analysis, each of the primary hypotheses will be tested using multiple imputations to replace the missing data under a variety of imputation distributions.

It is important to mention that our analysis will account for clustering at the clinic level as a random effect in all models. Although minimal, some variance in the SBP of participants could occur because the SBP of participants visiting the same physician could be correlated. Moreover, we are interested in the overall effect of the packaged intervention to see if it can eventually be rolled out as a policy across all primary-care practices. Therefore, we will not account for individual physician as a random effect in the main analysis.

However, we are collecting information on physicians at the clinic and will consider multilevel modeling with physicians as random effects in an additional analysis. This has been added in the revised manuscript.

#### Adverse events

Adverse events and serious adverse events will be recorded during the follow-up period and captured by the CRCs during the 4-monthly telephone follow-up calls or at the 1-year or 2-year visit by a CRC. Additionally, the nurses can also solicit information on adverse events every 4 weeks for the initial 3 months, and then every 3 months for the duration of the study during the telephone motivational advice sessions. Adverse events are categorized into one of several groups: angioedema and anaphylactic reaction, peripheral edema, hypotension, CHD, heart failure, stroke or transient ischemic attack, headache, dizziness or lightheadedness, flushing, cough after initiating antihypertensive medication, abdominal pain, muscle pain, falls and trauma, or other. Serious adverse events are defined as death, life-threatening events, events resulting in permanent disability, hospitalization, and prolongation of hospital stay.

#### Laboratory safety-monitoring tests

After high-risk CVD participants are identified and prescribed the SPC and statin, they are requested to come back about 4 weeks after their initial physician consultation to undergo safety monitoring for kidney, liver, and muscle related biomarkers that may become elevated after initiating these medications.

#### Economic evaluation

The primary purpose of this aim is to quantify the costs of the MCI from the societal, health system, and participant perspectives and determine its incremental cost-effectiveness ratio relative to usual care. Costs will be tracked using administrative records and standard cost collection instruments that capture all relevant labor, materials, and supplies, and participant out-of-pocket and other costs from an activity-based costing perspective. These instruments, which have been refined in prior hypertension trials, can identify key cost drivers of the intervention [16, 17]. Although we will track all costs, only incremental (non-sunk) costs will be used in the cost-effectiveness analysis, as these are the appropriate costs for determining whether to scale the intervention beyond the trial.

The government costs included in the cost-effectiveness analysis consist of non-sunk costs for training, the salaries of those delivering the intervention, and subsidies for outpatients, inpatients, diagnostic tests, and medications. Participant costs include out-of-pocket costs for medicines, health services, and transportation, and the dollar value of absenteeism and presenteeism, as measured by the Work Productivity and Activity Impairment Index and administered to patients at key assessment points. Incremental societal costs required to deliver the intervention include the dollar value of all payer and participant costs, including the dollar value of changes in productivity, that are above and beyond those incurred in the control arm.

#### Cost-effectiveness measures

1. Incremental cost per mmHg of SBP reduction from baseline to end of follow-up at 2 years post-randomization
2. Incremental cost per change in cardiovascular risk score from baseline to end of follow-up
3. Incremental cost per QALY gained based on responses to the EurolQol EQ-5D and published studies that convert changes in CVD risk scores to changes in lifetime QALYs
4. Incremental cost per DALY saved using the approach presented in Jafar et al. [18]

Each of these outcomes have been used in prior studies and can identify the benefits of the intervention relative to other hypertension trials and relative to standard benchmarks of cost-effectiveness [19, 20]. As part of the analysis, we will conduct one way and *n*-way sensitivity analyses and present cost-effectiveness acceptability curves to assess the impact of key model parameters and assumptions on the incremental cost-effectiveness ratios.

### Ethics

Ethical clearance will be obtained from the Central Institutional Review Board at SingHealth, which approved the pilot study.

### Study sample size

To compare the reduction in SBP at 2 years between the intervention and control groups, a sample size of 125 (subject to 20% attrition) in each of the eight polyclinics (four treatment and four control) would ensure at least 80% power to detect a difference between the treatment and control arms if the underlying difference between the arms is 0.28 standard deviations (Cohen’s effect size of 0.28) and the ICC of the SBP reductions within the same polyclinic is 0.016 or, more conservatively, if the underlying difference between the arms is 0.48 standard deviations (effect size of 0.48) and the intra-class correlation (ICC) of the SBP reductions within the same polyclinic is 0.05. Further, a sample size of 125 (subject to 20% attrition) in each of the eight polyclinics (four treatment and four control) would ensure at least 95% power to detect a difference between the treatment and control arms if the underlying difference between the arms is 0.36 standard deviations (effect size of 0.36) and the ICC of the SBP reductions within the same polyclinic is 0.01 or, more conservatively, if the underlying difference between the arms is 0.62 standard deviations (effect size of 0.62) and the ICC of the SBP reductions within the same polyclinic is 0.05.

An effect size of 0.3 is often regarded as a guideline for a clinically meaningful difference, while an effect size of 0.5 is often called a moderate effect. For SBP, a difference between the arms of 0.28 standard deviations might mean an absolute difference in differences between the treatment and control arms of 5 mmHg with a standard deviation of differences of 17.9 (5/0.28), or a difference in differences between the arms of 0.48 standard deviations might mean an absolute difference of 5 mmHg with a standard deviation of differences of 10.4 (5/0.48). Epidemiological evidence suggests that ICCs in excess of 0.05 are unlikely, and are more likely near 0.01, for objective measures such as a change in SBP in clusters of this size, as observed in the pilot data from four polyclinics.

The clinically meaningful detectable difference of 5 mmHg in SBP is consistent with our previous observations in COBRA, and sustained reductions in SBP of this magnitude are expected to lead to a 20% reduction in absolute risk of CVD events, a substantial benefit [21, 22]. At the same time, it is important to note evidence from trials of antihypertensive agents that smaller reductions in SBP (even 1 mmHg) decreased the risk of stroke by about 5% [23]. However, the main purpose of our trial is to demonstrate a clinically meaningful reduction in SBP and the cost-effectiveness of the intervention to convince policymakers and health planners to scale up the intervention. The latter is more likely with a sizable BP reduction.

## Discussion

Our proposed trial is novel in its comprehensive package of up-to-date potentially sustainable strategies targeting multiple risk factors with a focus on both BP and lipids in hypertensive individuals with uncontrolled hypertension visiting the public-sector polyclinics in Singapore. Additionally, the trial encompasses a monitor and evaluation methodology for the intervention’s effectiveness and cost-effectiveness. The major components include an emphasis on algorithm-based treatment using SPC antihypertensive medications as first-line agents and statins in all high-risk hypertensive individuals, motivational counseling strategies for enhancing adherence, task shifting to physician-supervised health workers, structured remote follow-up through telephone by nurses with a focus on cardiovascular risk reduction. The combination of motivational conversation on adherence to SPC and statins is likely to enhance further their uptake by the high-risk hypertensive individuals. Although SPC is discounted at 50% in the trial (e.g., the ARB/hydrochlorothiazide combination at S$3.2/week), it is relatively costly compared to the price of the two single antihypertensive agents available in the polyclinics (subsidized cost of S$2.8/week for both). This is where the motivational conversation is expected to help convince hypertensive individuals on the merits of two pills in one, such as fewer side effects, fewer pills, and hypertension control, in a system where part of the medication cost is still out of pocket. The strategy will be complemented by telephone follow-ups, which will further reinforce the desirable behaviors, leading to improved BP and reduction in cardiovascular risk. Although our trial is of relatively short duration and is not powered to assess the hard outcomes of CVD morbidity and mortality, the epidemiological and trial evidence have established that both reductions in BP and lipids are strongly correlated with CVD. Furthermore, a major strength of our trial is that both proposed primary and secondary outcomes are known to modify CVD risk substantially.

The cluster design with randomization at the polyclinic level greatly reduces the chance of contaminating the intervention across randomized groups and permits an unbiased evaluation of strategies at the health system level in Singapore. The randomized aspect of the study design also allows for an equal distribution of known and unknown confounders to the intervention and usual care arms. In addition, the detailed economic evaluation will provide valuable information about the financial viability of the model to policymakers. Thus, a demonstration of a sustainable hypertension control program that is implementable in busy polyclinics, provides an effective reduction of BP and cardiovascular risk, and is cost-effective relative to existing services would provide compelling evidence for upscaling the program across all primary-care centers in Singapore. The trial design ensures that each of the four components of the intervention can be integrated into the existing set-up of the polyclinic infrastructure and is feasible for implementation and translation in Singapore. For example, the training of providers will occur within the scheduled continued medical education lunch hour sessions, motivational conversation training will be delivered at the work site by specialists, and the choice of single-pill antihypertensive medications is based on agents already approved and available in the public-sector setting. Although there is redistribution of effort and a somewhat greater role of nurses in the telephone-based follow-up, the interventions will be delivered by the existing cadres of providers without creating a parallel system for delivering care.

Moreover, a project of this nature funded by republic sources would bring the hypertension management agenda into national focus. The economic analysis would also serve as a template for the projected cost-effectiveness of similar programs in other countries. We will further catalyze the process of research to policy translation in the Asia Pacific region by involving the key stakeholders from national governments, medical research councils, non-governmental organizations, and international agencies. Thus, our proposed trial has significant clinical practice and public health policy implications in Singapore and globally.

### Trial status

Participants are currently being recruited. The first patient was enrolled on 18 January 2017. The SPIRIT timeline for the study is shown in Table 1.

**Table 1:** Study timeline

	2016			2017				2018				2019			
	Q2	Q3	Q4	Q1	Q2	Q3	Q4	Q1	Q2	Q3	Q4	Q1	Q2	Q3	Q4
Protocol development	X														
Training of research coordinators and physicians	X		X	X				X							
Training of nurses for motivational counseling (yearly refresher training)		X			X				X						
Obtaining informed consent, recruitment, and baseline interview				X	X	X	X								
Auditing and site visit monitoring (every 2 weeks)				X	X	X	X	X	X	X	X	X	X	X	X
Trial supervision			X				X				X				X
Monthly Trial Management Committee meeting				X	X	X	X	X	X	X	X	X	X	X	X
Telephone follow-up by nurses (monthly until 3 months and then every 3 months)				X	X	X	X	X	X	X	X	X	X	X	X
Follow-up by clinical research coordinators (every 4 months by telephone; annually on site)					X	X	X	X	X	X	X	X	X	X	X
Data Safety and Monitoring Board meeting (every 6 months)		X			X		X		X		X		X		X
Trial Steering Committee Meeting (every 6 months)		X		X		X		X		X		X		X	
Quality assessment			X			X			X			X			X
Progress or interim report						X									
Data analysis														X	X
Manuscript writing														X	X
Final report														X	X

#### Trial governance and management

The chief principal investigator (THJ) will be responsible for the overall management of the trial, chairing the Trial Management Committee meeting, and conducting two audits at each clinic weekly. The day-to-day management at each site will be the responsibility of the eight site principal investigators. The study management group will comprise the chief principal investigator, main co-principal investigator (NCT), statistician (JA), site principal investigators, data managers, project coordinators, and research assistants. A Data Safety and Monitoring Board (DSMB) independent of the trial has been established as per MRC guidelines to review quality and safety issues. The DSMB operates in line with the MRC terms of reference as amended and agreed on by members at their first meeting.

### Dissemination plan

The results of the trial will be published in scientific journals and other media, and shared with key stakeholders in Singapore including administrative health officials of SingHealth and other health-care networks, professional organizations (cardiac, nephrology, and hypertension societies) and the Ministry of Health, and presented in local, regional, and international conferences. A health policy forum will be conducted to share the key findings in Singapore and regionally. The dissemination of the results is likely to enhance the scale-up of the trial strategies.

## Additional files

Additional file 1: SPIRIT checklist. (DOC 123 kb)

Additional file 2: Treatment algorithm. (PDF 121 kb)

Additional file 3: Physician management checklist. (DOCX 20 kb)

Additional file 4: Telephone follow-up checklist. (DOCX 30 kb)

## Abbreviations

ACEI: Angiotensin-converting-enzyme (ACE) inhibitor
ARB: Angiotensin II receptor blocker
BP: Blood pressure
CHD: Coronary heart disease
CRC: Clinical research coordinator
CVD: Cardiovascular disease
DALY: Disability adjusted life year
DBP: Diastolic blood pressure
DSMB: Data Safety and Monitoring Board
MCI: Multicomponent intervention
MRC: U.K. Medical Research Council
QALY: Quality adjusted life year
SBP: Systolic blood pressure
SPC: Single-pill combination
SPIRIT: Standard Protocol Items: Recommendations for Interventional Trials
