# Supplementary material for: Management of hypertension and multiple risk factors to enhance cardiovascular health in Singapore: The SingHypertension cluster randomized trial
Source: Trials. 2018 Mar 14;19:180. doi: 10.1186/s13063-018-2559-x (PMC5852962; doi:10.1186/s13063-018-2559-x)
Supplement: Supplementary file 2 — Treatment algorithm. (PDF 121 kb) [file 13063_2018_2559_MOESM2_ESM.pdf]

SingHypertension Study

Treatment Algorithm for Antihypertensive and Lipid Medications for Hypertensive Individuals with Uncontrolled Blood Pressure

Note: This treatment algorithm is **not applicable** for participants with any of the following conditions:

1) Unstable heart failure (EF<20%)
2) Advanced kidney disease defined as eGFR <40ml/min/1.73 m^2
3) Nephrotic range proteinuria (i.e. 3g/d or more)
4) Known advanced liver disease (i.e. Child-Pugh stage C)

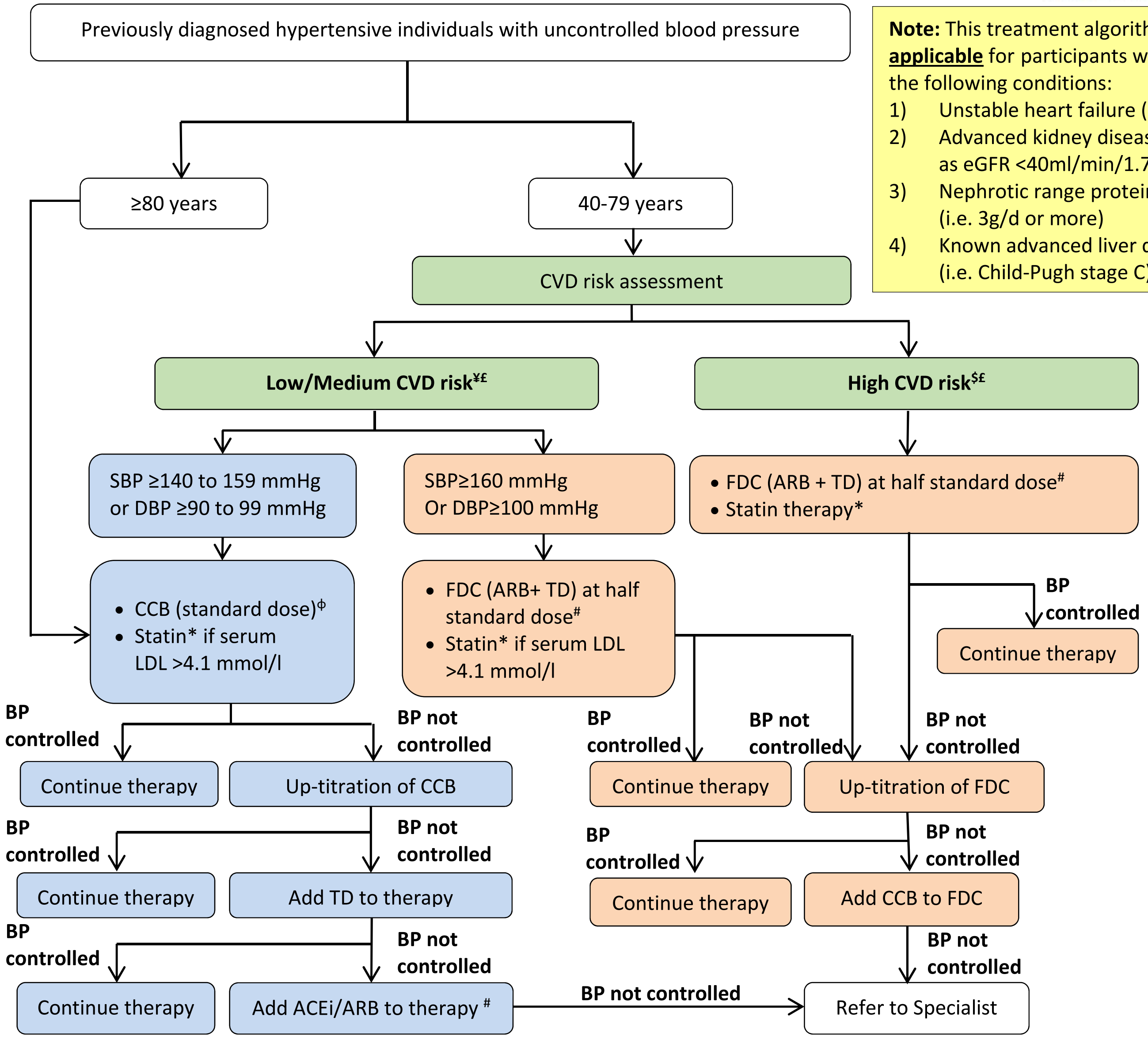

£ Individuals with compelling indications for beta-blocker (heart failure, post-myocardial infarction) are managed accordingly

ᶲ Continue current regimen if individuals are on ACEi/ARB or TD. Switch to CCB if on beta-blocker and uncontrolled BP

# Individuals initiated on ACEi/ARB will be given a laboratory request for measurement of serum sodium, potassium) and serum creatinine in 4-6 weeks.

* All high risk individuals will be treated with statin standard dose. Individuals started on statin will be given a request for serum alanine transaminase and muscle creatine kinase in 4-6 weeks.

CCB, calcium-channel blocker; **ACEi**, Angiotensin converting enzyme inhibitor; **ARB**, Angiotensin receptor blocker; **SBP**, Systolic blood pressure; **DBP**, Diastolic blood pressure; **FDC**, Fixed dose combination; **TD,** Thiazide-like diuretics; **Cr**, Creatinine; **ACR**, Albumin-to-creatinine ratio

¥ **Low/Medium CVD risk**
Individuals with CVD score identifying CVD risk <20% over 10 years without diabetes or previous history of CVD, or target organ damage, or renal disease

$ **High CVD risk**
Individuals with any one of below:

- CVD score identifying CVD risk ≥ 20% over 10 years
- Diabetes
- Target organ damage
  1. Left ventricular hypertrophy - *as evidenced by echocardiography or electrocardiography if individual has one*
  2. Retinopathy
  3. Proteinuria - *ACR 34 mg/mmol; 300 mg/day or more of albumin excretion*
  4. Renal disease *(Estimated GFR <60 ml/min/1.73m^2)*
- Pre-existing CVD *(previous history of heart disease, stroke)*

Document version date 1 Dec 2016
