# Supplementary material for: Management of hypertension and multiple risk factors to enhance cardiovascular health in Singapore: The SingHypertension cluster randomized trial
Source: Trials. 2018 Mar 14;19:180. doi: 10.1186/s13063-018-2559-x (PMC5852962; doi:10.1186/s13063-018-2559-x)
Supplement: Supplementary file 3 — Physician management checklist. (DOCX 20 kb) [file 13063_2018_2559_MOESM3_ESM.docx]

**Management of Hypertension and multiple risk factors to Enhance Cardiovascular**

**Health – A Cluster Randomized Trial in SingHealth Polyclinics, Singapore**

**CHECKLIST TO IDENTIFY HIGH CVD RISK SUBJECTS BY PRIMARY CARE PHYSICIANS**

| 1 | Subject ID number |  |
| --- | --- | --- |
| 2 | Date (dd/mm/yyyy) | _ _ / _ _ / _ _ _ _ |
| 3 | Age of the participant |  |
| 4 | Signature of Physician |  |
| 5 | Clinic code |  |

| **Checklist to Identify High CVD Risk Subjects** | | | | |
| --- | --- | --- | --- | --- |
| **No.** | **Item** | **Yes** | **No** | **Remark** |
| 1 | Does participant have a CVD score identifying CVD risk **≥20%** over 10 years? | □ | □ |  |
| 2 | Does participant have **Diabetes**? | □ | □ |  |
| 3 | Does participant have **Left Ventricular Hypertrophy** (as evidenced by echocardiography or electrocardiography if participant has one)? | □ | □ |  |
| 4 | Does participant have **retinopathy**? | □ | □ |  |
| 5 | Does participant have **proteinuria** [ACR >34 mg/mmol of Cr; Urine albumin excretion >300 mg/day]? | □ | □ |  |
| 6 | Does participant have **renal disease** (Estimated GFR <60 ml/min/1.73m^2^)? | □ | □ |  |
| 7 | Does participant have **previous history of heart disease**? | □ | □ |  |
| 8 | Does participant have **previous history of stroke**? | □ | □ |  |

**CVD**, Cardiovascular Disease; **ACR**, Albumin-to-creatinine ratio; **Cr**, Creatinine

1. If any one of above items is “Yes”, then the participant belongs to high CVD risk group. Otherwise, s/he belongs to low/medium CVD risk group.

Based on the information above, the study participant has: **(Tick one below)**

□ **High CVD risk – skip to question B**  □ **Low/medium CVD risk – skip to question D**

1. If the participant belongs to High CVD risk group and falls in the age group 40-79, then was s/he prescribed fixed dose combination (FDC) drug and statin as per the study treatment algorithm? **(Tick one below)**

□ **Yes -> END**  □ **No – skip to question C**

1. If no, then what was the reason for not prescribing the FDC?

_________________________________________________________________________________

­­­­­­­ _________________________________________________________________________________

_________________________________________________________________________________

1. If the participant belongs to Low/medium CVD risk group having uncontrolled BP (SBP ≥160mm Hg or DBP ≥100 mm Hg) and falls in the age group 40-79, then was s/he prescribed fixed dose combination (FDC) drug and statin (only if serum LDL >4.1 mmol/l) as per the study treatment algorithm? **(Tick one below)**

□ **Yes -> END**  □ **No – skip to question E**

1. If no, then what was the reason for not prescribing the FDC?

_________________________________________________________________________________

­­­­­­­ _________________________________________________________________________________

_________________________________________________________________________________
