# Supplementary material for: Management of hypertension and multiple risk factors to enhance cardiovascular health in Singapore: The SingHypertension cluster randomized trial
Source: Trials. 2018 Mar 14;19:180. doi: 10.1186/s13063-018-2559-x (PMC5852962; doi:10.1186/s13063-018-2559-x)
Supplement: Supplementary file 4 — Telephone follow-up checklist. (DOCX 30 kb) [file 13063_2018_2559_MOESM4_ESM.docx]

**Management of Hypertension and multiple risk factors to Enhance Cardiovascular Health – A Cluster Randomized Trial in SingHealth Polyclinics, Singapore**

**CHECKLIST FOR TELEPHONE FOLLOW-UP BY NURSES**

***Instructions:*** *At least 3 attempts should be made to make a telephone call in case of “No Answer” from the participant. Please fill the following information in case of lost-to follow-up:*

| **Attempt number** | **Date (dd/mm/yyyy)** | **Time (hh:mm)** |
| --- | --- | --- |
| 1 |  |  |
| 2 |  |  |
| 3 |  |  |

***Instructions:*** *If the telephone call is answered then introduce yourself and mention the name of the clinic from where you are calling. Remind the participant about the purpose of the call and time required to answer the questions. If agreed proceed to section A, else make an appointment for another call as per the participant’s convenience and enter details below:*

| **Appointment details** | |
| --- | --- |
| **Preferred date (dd/mm/yyyy)** | **Preferred time (hh:mm)** |
|  |  |

| **Section A: Details of telephone follow-up** | | |
| --- | --- | --- |
| **No.** | **Item** | **Response** |
| A1 | Name of the nurse clinician or nurse who conduct the telephone follow-up |  |
| A2 | Date (dd/mm/yyyy) | _ _ / _ _ / _ _ _ _ |
| A3 | Interview start time (hh:mm) |  |
| A4 | Interview end time (hh:mm) |  |
| A5 | Baseline BMI |  |
| A6 | Follow-up number | □ 1^st^ follow-up (after 1 month of enrolment)  □ 2^nd^ follow-up (after 2 months of enrolment)  □ 3^rd^ follow-up (after 3 months of enrolment)  □ 4^th^ follow-up (after 6 months of enrolment)  □ 5^th^ follow-up (after 9 months of enrolment)  □ 6^th^ follow-up (after 12 months of enrolment)  □ 7^th^ follow-up (after 15 months of enrolment)  □ 8^th^ follow-up (after 18 months of enrolment)  □ 9^th^ follow-up (after 21 months of enrolment)  □ 10^th^ follow-up (final follow-up) |
| A7 | Clinic code |  |

| **Section B: Home Blood Pressure Monitoring** | | | | |
| --- | --- | --- | --- | --- |
| **No.** | **Item** | **Yes** | **No** | **Remark** |
| B1 | Do you own a Blood Pressure monitor?  ***(If no, go to item C1 and advise to use home BP monitor)*** | □ | □ |  |
| B2 | Do you measure your BP at home regularly? | □ | □ |  |

| **Section C: Lifestyle modification for control of hypertension and global cardiovascular risk** | | | | | |
| --- | --- | --- | --- | --- | --- |
| **No.** | **Item** | **Aim** | **Yes** | **No** | **Remark** |
| C1 | Have you been having low salt diet? | Low salt diet | □ | □ |  |
| C2 | Have you been having a diet high in fruits and vegetables? | High content of fruits and vegetables in diet | □ | □ |  |
| C3 | Did you exercise regularly since our last conversation? | Exercise for 30 minutes for 5 or more days per week. | □ | □ |  |
| C4 | Did you drink excessively since our last conversation? | No more than 2 standard drinks per day 1 standard drink is   - 2/3 small can of beer (220 ml) - 1 glass of wine (100ml) - 1 nip of spirit (30ml) | □ | □ |  |

| C5 | Did you smoke since our last conversation? | Stop smoking | □ | □ |  |
| --- | --- | --- | --- | --- | --- |
| C6 | ***For patients with BMI ≥ 23.5 at baseline***, have you been trying to lose weight since our last conversation? | BMI < 23.5 | □ | □ |  |

| **Section D** | | | | | |
| --- | --- | --- | --- | --- | --- |
| **Hypertension medication adherence** | | | | | |
| **No.** | **Item** | | **Yes** | **No** | **Remark** |
| D1 | Was there any missed dose for your Hypertension medication since our last conversation?  **(If yes, go to next item D2. If No, go to D5.)** | | □ | □ |  |
| D2 | ***If Yes***, how many times did you miss Hypertension medication? | | | | Number of times: ______ |
| D3 | If poor adherence, what are the reasons? **(Tick all that apply)**  ***(If medication side effects, notify study coordinator for completing adverse events reporting form)*** | Medication Side effects | | □ |  |
|  |  | Forgetfulness | | □ |  |
|  |  | Misunderstanding about following regimen | | □ |  |
|  |  | Changing schedule | | □ |  |
| D4 | Other reasons, if any: | | | | |
| **Lipid medication adherence** | | | | | |
| **No.** | **Item** | | **Yes** | **No** | **Remark** |
| D5 | Was there any missed dose for your Lipid medication since our last conversation?  **(If yes, go to next item D6. If No, go to section E.)** | | □ | □ |  |
| D6 | ***If Yes***, how many times did you miss Lipid medication? | | | | Number of times: ______ |
| D7 | If poor adherence, what is the reason? **(Tick all that apply)**  ***(If medication side effects, notify study coordinator for completing adverse events reporting form)*** | Medication Side effects | | □ |  |
|  |  | Forgetfulness | | □ |  |
|  |  | Misunderstanding about following regimen | | □ |  |
|  |  | Changing schedule | | □ |  |
| D8 | Other reasons, if any: | | | | |

| **Section E** | | | | |
| --- | --- | --- | --- | --- |
| **Action** | | | | |
| **No.** | **Item** | **Reinforced the advice on:** | | **Remark** |
| E1 | Action taken during the telephone follow-up: | Advice on lifestyle modification strengthened | □ |  |
|  |  | Advice on medication adherence strengthened | □ |  |
|  |  | Medication side effect recorded | □ |  |
|  |  | Advice on home BP monitoring strengthened | □ |  |
| E2 | Other actions taken, if any: |  |  | |
| **Communication with Physician** | | | | |
| E3 | Additional action**,** if any, following the discussion with physician |  | | |
| E4 | Name and signature of the physician | **Name: ________________** **Signature: _______________**  **Date (dd/mm/yyyy): _ _ / _ _ / _ _ _ _** | | |
| E5 | Further communication with the participant following the discussion with physician |  | | |
| E6 | Dated signature of the nurse clinician or nurse after completion of actions | **Signature: _______________**  **Date (dd/mm/yyyy): _ _ / _ _ / _ _ _ _** | | |
